# Evaluation of an updated educational intervention on nutritional care to prevent undernutrition among older adults in primary health care

**DOI:** 10.1017/S1463423624000690

**Published:** 2025-01-24

**Authors:** Erika Berggren, Christina Sandlund, Liisa Samuelsson, Lena Lundh

**Affiliations:** 1 Academic Primary Health Care Centre, Stockholm County Council, Sweden; 2 Department of Neurobiology, Care Sciences and Society; Karolinska Institutet, Huddinge, Sweden

**Keywords:** caring, collaboration, learning, responsibility, undernourishment

## Abstract

**Aim:**

The aim of this study was to evaluate district nurses’ perceived and factual knowledge about nutritional care after an updated and expanded educational intervention. Furthermore, we aimed to compare the outcomes of the revised and the original educational intervention.

**Background:**

In-depth knowledge of nutritional care is a prerequisite to supporting older adults’ well-being and health. District nurses’ actual knowledge of the nutrition care process, older adults’ need for food, and palliative care in diverse phases of disease is therefore of utmost importance. An updated and expanded educational intervention meeting these needs was evaluated.

**Methods:**

A study-specific questionnaire about nutritional care was used before and after the educational intervention. Participants (*n* = 118) were district nurses working in primary health care in Region Stockholm. Additionally, a pre- and post-test quasi-experimental design was used to assess differences in learning outcomes of the revised intervention compared with the original intervention.

**Findings:**

District nurses who completed the questionnaire had worked in health care for about 18 years and as district nurses for 5 years after their specialist examination. After the revised educational intervention, significant improvements were found in all statements concerning perceived challenges and actions related to nutritional care, while questions about factual knowledge showed significant improvements in three of the four questions.

Comparison between the revised and the original intervention revealed no differences in most areas of perceived challenges and actions related to nutritional care. Additionally, in half of the areas assessed, factual knowledge improved more after the revision than after the original educational intervention, including the maximum length of overnight fast and the type of oral nutritional supplements (ONS) that should be prescribed.

**Conclusion:**

The intervention was successful in increasing knowledge about nutritional care, nutritional counselling, food adaptation, and prescribing ONS in an individually tailored way. In-depth knowledge supports usability in clinical practice. Nevertheless, we need to follow-up and understand how increased knowledge about undernutrition and ONS prescription are implemented in primary health care when caring for older adults’ desires and needs.

## Introduction

In primary health care, both physicians and nurses are responsible for recognizing older patients’ nutritional needs and providing nutritional care (Keller *et al.*, 2022; National Board of health and Welfare, 2020). However, older patients’ nutritional needs and risk for undernutrition are often missed and underprioritized (Volkert, 2013). One reason may be a need to provide more education for healthcare professionals, so they are better qualified to provide adequate nutritional care (Volkert *et al.*, 2019; Lindner-Rabl *et al.*, 2022). Other potential reasons include a lack of awareness by primary healthcare personnel regarding their professional responsibilities (Fjeldstad *et al.*, 2018; Dominguez Castro *et al.*, 2020), as well as unclear division of responsibilities at the workplace (National Board of health and Welfare, 2017). When patients’ risk for undernutrition goes unrecognized, it can intensify fatigue and depression (Baldwin *et al.*, 2016), worsen disease, increase suffering (Gomez-Batiste *et al.*, 2017), and even hasten death (Söderström *et al.*, 2017).

European Society for Clinical Nutrition and Metabolism (ESPEN) points out that one barrier to proper nutritional support may be a lack of sufficient education in nutritional care aimed at health care professionals (Volkert *et al.*, 2022). However, results from educational interventions concerning nutritional care appear to focus on nurses’ education of patients and caregivers (Smith *et al.*, 2020; Ten Cate *et al.*, 2020). Furthermore, educational interventions about nutrition often include more about treatments for specific conditions, such as dementia (Jackson *et al.*, 2011), stroke, and dysphagia (Labeit *et al.*, 2024), and less about the patient’s situation from a more holistic perspective. It has also been found that almost half of the nurses in home health care report that they did not screen for malnutrition (Cate ten *et al.*, 2021). Previous studies from Cate ten et al. suggest that nurses need more training on malnutrition to enhance the nutritional care of older adults throughout the care continuum (Cate ten *et al.*, 2021; Cate *et al.*, 2022). Moreover, support for educational interventions that are adapted to this context, aimed at in-depth learning that is useful in practice, is rare.

Several educational interventions for health care professionals take support from the pedagogical theory, constructive alignment, by John Biggs (Biggs, 1996; Biggs *et al.*, 2022) to enhance learning by aligning learning objectives, activities, and follow-up evaluations. To further adapt this approach to the context of nutritional care, a three-part continuing education model for primary health care professionals (ConPrim) (Berggren *et al.*, 2016) was devised to increase in-depth knowledge with benefits for practice.

The intervention in the current study is an updated and expanded (hereafter “revised”) version of an intervention developed in 2011 by the authors at the request of the Stockholm County Health and Medical Care Administration. The original intervention, described in detail in a previous publication (Samuelsson *et al.*, 2019), resulted in significant improvements in district nurses’ perceived and factual knowledge about nutritional care, but also identified areas in which knowledge could be further improved. Over time, district nurses have gained responsibility for more patients with advanced care needs, such as palliative care and nutritional care (Berggren *et al.*, 2019; World Health Organization, 2015). In addition, the Sweden National Board of Health and Welfare published revised guidelines for palliative care and for nutritional care (National Board of health and Welfare, 2017; National Board of Health and Welfare, 2013). It was, therefore, apparent that a revised intervention was needed.

## Aim

We aimed to evaluate district nurses’ perceived and factual knowledge about nutritional care after an updated and expanded educational intervention. In addition, we aimed to compare the outcomes of the revised and the original educational intervention.

## Methods

### Study design, setting, and participants

This study had a pre-test and post-test quasi-experimental design (Polit & Beck 2021). Data were gathered using a study-specific questionnaire before and after the educational intervention.

Participants in the revised intervention were district nurses working at the approximately 200 primary health care centres in Region Stockholm. During the study period (2017–2019), 132 district nurses started and 129 completed the revised intervention. A total of 118 of the 129 (91%) responded to the questionnaire before the course started and after it ended. Reasons for not completing the questionnaire included arriving late on the first day or leaving early on the last day. Seventy-four of the 118 respondents (63%) worked full or part-time within home health care. Participants in the original intervention included 456 district nurses and the majority worked, at least partly, in home health care (Samuelsson *et al.*, 2019).

### The intervention

The continuing educational intervention (hereafter the educational intervention) evaluated in the current study was designed to increase district nurses’ knowledge about nutritional care and thereby prevent undernutrition in older adults. A course titled ‘Prevent undernutrition and prescribe oral nutritional supplements correctly’ is offered twice a year by Region Stockholm Academic Primary Health Care Centre. This course, carried out over three months, consists of interactive lectures and a practical exercise conducted in clinical practice with a follow-up group discussion in a later course session (Table 1). Two and a half days are spent in the classroom. Passing this course gives district nurses the right to prescribe oral nutritional supplements (ONS) in Region Stockholm.

**Table 1.** Content of the revised educational intervention

Session 1	
	Prevention and treatment of undernutrition*
	Disease-related undernutrition
	Food-related causes of undernutrition
	Assessing nutritional needs and the need for oral nutritional supplements*
	Nutritional care*
	Documentation of nutritional care in electronic patient records**
Between sessions	
	Practical exercise in nutritional care, patient case conducted in own clinical practice, documented in a written assignment
Session 2	
	Follow-up discussions, in small groups, with feedback of patient cases*
	Guidelines and how to prescribe oral nutritional supplements via Region Stockholm’s electronic portal
	Oral nutritional supplements approved for use in Region Stockholm and choosing which supplement to prescribe
	Adjusting the consistency of food
	Tasting oral nutritional supplements
	Collaboration and responsibility for nutritional care
	Presentation and discussion of patient cases in small groups
Between sessions	
	Written feedback on and revision of patient case*
	Self-study via a web program on nutritional care for patients who have home health care, including their needs in palliative phases of disease***
Session 3	
	Group discussion on taking a palliative approach to care and nutritional care to meet patients’ needs in early and late palliative stages of disease (based on the web program)***
	Follow-up seminar on responsibility for nutritional care for patients in primary health care: reflection based on factual knowledge gained during the course and professionals’ previous knowledge and skills
	Follow-up group discussion of written assignment: nutritional care for a patient in own clinical practice (patient case)*

*More in-depth information on this topic or feedback on the activity was added in response to the results of the original evaluation.

**Time spent on this topic was reduced to compensate for the need to provide more information on other topics.

***New activity (added to the revised intervention).

The revised intervention included changes made in response to the results of the original evaluation (Samuelsson *et al.*, 2019) and updated guidelines. These changes aimed to further improve the nurses’ perceived and factual knowledge about nutritional care and their confidence in that knowledge. In accordance with the pedagogical constructive alignment theory, intended learning outcomes, teaching and learning activities, and follow-up assessment were aligned to enable participants to achieve deeper levels of knowledge (Biggs *et al.*, 2022). Further adaptations were made using the ConPrim model (Berggren *et al.*, 2016), and increased emphasis was placed on reflective small- and large-group discussions. The dietitian (specialist in the nutritional care process) lectured in greater detail about energy and fluid requirements and the types of ONS that are appropriate for patients’ individual needs. The district nurses received more comprehensive feedback on the written assignment that was included in the practical exercise and submitted written revisions and responses to the feedback.

Since frail older adults are increasingly cared for at home by primary health care professionals, the updated intervention included information about patients’ nutritional needs in different palliative phases of disease and the importance of collaboration. The new information was provided in a web-based program that the district nurses completed independently. Subjects important to discuss were the primary health care assignment, responsibilities, conversation at transition points, and the importance of interprofessional collaboration in meeting these advanced needs. The web-based program presented facts about the topics and included a short printable introduction. Descriptive drawings illustrated a patient case, representative of those seen in primary health care, followed by a patient case and interactive multiple-choice questions to assess what the participant had learned. When participants had answered all the questions correctly, which was one of the requirements for passing the course, they received a certificate. At the final course seminar, the nurses then took part in follow-up discussions about the content of the web-based program and how it related to their own experiences.

The participants were expected to be able to identify and assess risk for undernutrition and nutrition-related symptoms, distinguish nutritional needs in early and late palliative phases of disease, and plan for and follow-up individual needs regarding food and nutrition. They should be able to apply a variety of working methods to provide good nutritional care, including prescribing oral nutritional supplements correctly when dietary counselling alone is insufficient. Participants were also expected to be able to evaluate the results of the care interventions.

### Data collection

To measure changes in perceived and factual knowledge about the areas of nutritional care covered in the educational intervention, a study-specific questionnaire was distributed to participants in the classroom prior to the start of the first-course session and at the end of the final session.

#### The questionnaire

As described in a previous publication, researchers from the Academic Primary Health Care Centre developed the questionnaire. Sixteen items from the questionnaire were included in the evaluation of the original intervention (Samuelsson *et al.*, 2019). For evaluation of the revised intervention, four new statements concerning the updated and expanded parts of the course were added to the questionnaire (about food and palliative phase, conversations at transition points, collaboration, and meal environment). For brevity, two items from the demographic and background section were removed, as was a question concerning use of documentation.

The revised questionnaire included 17 statements or questions that covered the district nurses’ background (3 questions), perceived challenges and actions related to nutritional care (10 statements, Table 2), and factual knowledge (4 questions, Table 3). The statements all had Likert-type response alternatives: ‘fully agree’ (score = 1), ‘mainly agree’ (score = 2), ‘partly agree’ (score = 3), and ‘do not agree at all’ (score = 4). All the questions were multiple choice; each question had between five and eight response alternatives, of which one was correct.

**Table 2.** Responses to statements about perceived challenges and actions related to nutritional care before and after the revised intervention (matched pairs) (n = 118)

		Responses				Change	
Statements		Fully Agree n (%)	Mainly agree n (%)	Partly agree n (%)	Do not agree at all n (%)	Mean (SD) Median (IQR)	*P* value*
I find it difficult to							
… identify patients who are undernourished or at risk for undernutrition (n = 118)^1^	Before	3 (2.5)	19 (16.1)	84 (71.2)	12 (10.2)	2.89 (0.6) 3.0 (3-3)	< 0.001
	After	2 (1.7)	7 (5.9)	80 (67.8)	29 (24.6)	3.15 (0.6) 3.0 (3.0–3.25)	
… know what actions I should take for patients who are undernourished or at risk for undernutrition (n = 118)^1^	Before	5 (4.2)	29 (24.6)	77 (65.3)	7 (5.9)	2.73 (0.6) 3.0 (2.0–3.0)	< 0.001
	After	0 (0)	3 (2.5)	58 (49.2)	57 (48.3)	3.46 (0.5) 3.0 (3.0–4.0)	
… choose which ONS it is appropriate to prescribe for each individual patient (n = 117)^1^	Before	28 (23.9)	50 (42.7)	37 (31.6)	2 (1.7)	2.11 (0.8) 2.0 (2.0–3.0)	< 0.001
	After	1 (0.8)	12 (10.2)	84 (71.2)	21 (17.8)	3.06 (0.6) 3.0 (3.0-3.0)	
… know how food should be adapted to the palliative phase the patient is in (for example, with regard to fat and protein) (n = 118)^1^	Before	43 (36.4)	48 (40.7)	26 (22.0)	1 (0.8)	1.87 (0.8) 2.0 (1.0–2.0)	< 0.001
	After	5 (4.2)	25 (21.2)	66 (55.9)	22 (18.6)	2.89 (0.7) 3.0 (2.0–3.0)	
… know when to have a conversation with the patient about critical transition points (n = 116)^1^	Before	37 (31.9)	45 (38.8)	27 (23.3)	7 (6.0)	2.03 (0.9) 2.0 (1.0–3.0)	< 0.001
	After	1(0.9)	24 (20.7)	58 (50.0)	33 (28.4)	3.06 (0.7) 3.0 (3.0–4.0)	
… collaborate with other health care providers about patients with malnutrition or risk for malnutrition (n = 117)^1^	Before	10 (8.5)	32 (27.4)	55 (47.0)	20 (17.1)	2.73 (0.8) 3.0 (2.0–3.0)	< 0.001
	After	2 (1.7)	18 (15.3)	69 (58.5)	29 (24.6)	3.06 (0.7) 3.0 (3.0-3.25)	
I always conduct a dietary assessment when I suspect undernutrition (24-hour recall) (n = 117)^2^	Before	8 (6.8)	29 (24.8)	56 (47.9)	24 (20.5)	2.82 (0.8) 3.0 (2.0–3.0)	< 0.001
	After	41 (35.0)	25 (21.4)	43 (36.8)	8 (6.8)	2.15 (1.0) 2.0 (1.0–3.0)	
I calculate BMI for all new patients in home health care (n = 71)^2^ ^,^^3^	Before	29 (40.8)	16 (22.5)	13 (18.3)	13 (18.3)	2.14 (1.2) 2.0 (1.0–3.0)	< 0.001
	After	39 (52.7)	16 (21.6)	15 (20.3)	4 (5.4)	0.35 (1.0) 0.0 (0.0–1.0)	
I regularly follow-up the weight of all home health care patients I am responsible for (n = 70)^2^ ^,^^3^	Before	30 (42.9)	21 (30.0)	17 (24.3)	2 (2.9)	1.87 (0.9) 2.0 (1.0–3.0)	< 0.001
	After	40 (55.6)	17 (23.6)	14 (19.4)	1 (1.4)	0.21 (0.8) 0.0 (0.0–1.0)	
As a district nurse in home health care, it is my responsibility to assess the patient’s dining area and meal environment (n = 71)^2^ ^,3^	Before	18 (25.4)	25 (35.2)	25 (35.2)	3 (4.2)	2.18 (0.9) 2.0 (1.0–3.0)	= 0.001
	After	34 (46.0)	27 (36.5)	12 (16.2)	1 (1.4)	1.71 (0.8) 2.0 (1.0–2.0)	

ONS, oral nutritional supplements; SD, standard deviation; IQR, interquartile range; BMI, body mass index.

*Wilcoxon signed rank test (two-sided).

1 “Do not agree at all” gave the highest score.

2 “Fully agree” gave the highest score.

3 Only those who worked in home health care answered this question (n = 74).

**Table 3.** Factual knowledge and number and percentage of correct answers before and after the revised intervention (n = 118)

	Before n (%)	After n (%)	Change *P* value*
What type of ONS would you prescribe for a patient who has difficulty swallowing and repeatedly had pneumonia? (n = 108)	50 (46.3)	88 (81.5)	0.000
What do you think is the energy requirement for an 82-year-old woman who can walk short distances with a walking frame but sits most of the day, eats independently, weight 60 kilos, and has a BMI of 24? (n = 114)	32 (28.1)	47 (41.2)	0.053
What do you think is the fluid requirement for an 82-year-old woman who can walk short distances with a walking frame but sits most of the day, eats independently, does not have a fever, weight 60 kilos, and has a BMI of 24? (n = 114)	28 (24.6)	43 (37.7)	0.036
How long should the maximum night fast be for older patients? (n = 116)	57 (49.1)	99 (85.3)	0.000

Abbreviations: ONS, oral nutritional supplements; BMI, body mass index.

*McNemar test, binomial distribution used.

### Data analysis

Analyses were performed using IBM SPSS (version 26, IBM Corp., Armonk, NY, USA). The significance level was 5% (two-tailed). Analyses were conducted on observed data. Variables were summarized with standard descriptive statistics, such as frequency, mean, standard deviation (SD), median, and interquartile range.

Differences in perceived challenges and actions related to nutritional care before and after the revised educational intervention were calculated for each participant using the Wilcoxon Signed-Rank Test. The McNemar Test (binomial distribution) was used to calculate differences in factual knowledge before and after the revised educational intervention.

The independent t-test was used to compare background characteristics of the group that received the revised educational intervention and the group that received the original educational intervention. The Mann–Whitney U test was used to calculate differences in change in perceived challenges and actions related to nutritional care in district nurses who had received the revised intervention and those who had received the original intervention, from baseline to follow-up (baseline outcomes minus follow-up outcomes for each participant). Pearson’s Chi-square test was used to investigate differences in the proportion of district nurses who responded correctly to the questions about factual knowledge before the revised intervention and the original intervention and after the revised intervention and the original intervention.

## Results

### Demographic and background information

The questionnaire was completed by 118 district nurses, mean age 43.7 years (SD 10.4), who had worked in health care for a mean of 18.2 years (SD 10.9), and as a district nurse for a mean of 5.1 years (SD 6.8).

### Perceived challenges and actions related to nutritional care

District nurses’ responses to the statements about perceived challenges and actions related to nutritional care show that they found it less difficult to provide nutritional care after the intervention (Table 2). The differences in median scores before and after the intervention were statistically significant for all statements, including the new statements about needs and the palliative phase, conversations at transition points, collaboration, and meal environment. For instance, the responses indicated that after the intervention, the district nurses found it easier to know how meals should be adapted to patients’ individual needs in different palliative phases of disease (*P* < .001).

### Factual knowledge

District nurses’ factual knowledge improved after the revised intervention (Table 3). Significant improvements were observed in knowledge about the type of ONS that should be prescribed, fluid requirements, and the maximum length of the overnight fast (Table 3).

### Comparison of the revised and the original intervention

District nurses who received the revised educational intervention were younger, had shorter health care experience, and had worked fewer years as district nurses than participants who received the original educational intervention (*P* = < .001) (Table 4).

**Table 4.** Comparison of background characteristics of participants who received the revised intervention and participants who received the original intervention

	Revised intervention (n = 118)		Original intervention (n = 459)		
Variable	n	mean (SD*)	n	mean (SD*)	*P* value**
Age in years	111	43.7 (10.4)	453	50 (8.4)	< 0.001
Years worked in health care	116	18.2 (10.9)	458	26.4 (10.4)	< 0.001
Years worked as a district nurse	114	5.1 (6.8)	457	10.5 (8.3)	< 0.001

*SD = standard deviation.

**independent t-test.

For six of the ten statements about perceived challenges and actions related to nutritional care, there were no differences in improvements following the revised and original interventions. However, district nurses’ uncertainty about identifying patients who are undernourished, or at risk for undernutrition, had reduced less among those who had participated in the revised intervention than those who participated in the original intervention (mean change from baseline to follow-up, revised intervention, -0.26 [SD 0.7] vs. original intervention, -0.49 [SD 0.8]; *P* = 0.003). The same pattern was observed in district nurses’ degree of agreement with the statement that they always conduct a dietary assessment when they suspect undernutrition (mean change from baseline to follow-up, 0.65 [SD 1.1] vs. 0.99 [SD 1.0]; mean rank 239.8; *P* = 0.001). Among district nurses who worked in home health care, the degree of agreement with the statement that they regularly follow-up on the weight of patients in home health care increased less after the revised than after the original intervention (mean change from baseline to follow-up, 0.21 [SD 0.8] vs. 0.60 [SD 1.0]; *P* = 0.004).

For two of the four questions that assessed factual knowledge, a higher percentage of district nurses who took part in the revised intervention responded correctly at follow-up: the type of ONS that should be prescribed for a patient who had difficulty swallowing and repeatedly had pneumonia (revised intervention 80.7% vs. original intervention 71.4%, *P* = 0.047) and maximum length of overnight fast (revised intervention 85.5% vs. original intervention 66.9%, *P* < 0.001). At baseline, factual knowledge about these two questions was similar in the two groups of district nurses (type of ONS, *P* = 0.960; length of night fast, *P* = 0.103).

## Discussion

Relevant knowledge concerning nutritional care and the ability to implement guidelines into the primary health care context are of utmost importance. This study evaluated a revised educational intervention that had been updated and expanded and, in summary, found improvements relating to the nutritional care of older adults with focus on their risk for undernutrition. After the educational intervention, district nurses’ assessment of perceived challenges and actions related to nutritional care improved significantly in all areas and the assessment of factual knowledge had improved in all four areas.

The added content concerning older adults’ needs in palliative care also included new learning activities, such as a web-based educational program about older adults’ need for food and palliative care in diverse phases of disease, which was followed up with discussions and reflection in the group (Berggren *et al.*, 2016). More in-depth information concerning older adults’ needs was given in this revised part of the course. This revision is of importance since older adults are currently living longer and with several incurable diseases. The added content is also in line with facts that show that more older people are frail and vulnerable to undernutrition (Gomez-Batiste *et al.*, 2017; Dent *et al.*, 2023; Volkert *et al.*, 2022). Additionally, in the transition to when the older adult can no longer eat or assimilate food or nourishment, the nutritional goal needs to be updated and comfort feeding should be offered (Volkert *et al.*, 2022). In our intervention, the meaning of comfort feeding was clarified and district nurses’ perception of how to adapt food to older adults’ needs in diverse phases of disease showed significant improvements.

The results in the current study reveal a high awareness of nutritional care among district nurses and abilities that are important in preventing undernutrition, prescribing nutritional supplements in an individually tailored way, and meeting patients’ needs in diverse phases of disease. In our revised intervention, the education was expanded with more interactive seminars, self-studies via e-learning, and more time for the practical exercise (Berggren *et al.*, 2016). In-depth feedback and time for small-and large-group reflective discussions were included to facilitate participants’ interaction and learning, all supported by the pedagogical theory, constructive alignment (Biggs, 1996; Biggs *et al.*, 2022). The parts were aligned to enable participants to achieve deeper levels of learning and understanding of the improvement of nutritional care in clinical practice. To ensure alignment with primary health care, the seminar discussions were facilitated by authentic patient cases.

Considering the need for education to provide suitable nutritional care intended for health care professionals, two diverse strategies for education have been compared (Törmä *et al.*, 2021). It was found that the educational intervention using external facilitation (which was longer) had a more active educational approach and increased the participants’ ability to implement guidelines in nutritional care, compared to the group receiving an intervention with a shorter and more passive educational approach (Törmä *et al.*, 2021). Similar results were found in the present study where the district nurses learned more in-depth about nutritional counselling, food adaptation, and how to prescribe ONS in an individually tailored way. They also calculated BMI for new patients in home health care and followed patients’ body weight in home health care more frequently. In the educational intervention, we counselled that all older adults should be screened for undernutrition to identify risk early and, together with the patient, discuss and adapt actions for nutritional care. Our findings indicate that active participation in discussions seems to increase knowledge about important subjects, such as the meaningful conversation at transition points (Åvik Persson *et al.*, 2023). Moreover, the meaning of a holistic view and a person-centered approach (McCormack and McCance, 2017; World Health Organization WHO, 2002; Swedish regions in collaboration, 2023) was raised in group discussions in relation to district nurses’ work and experiences in caring for older adults and their desires based on their life situation.

We also found that the district nurses significantly increased their factual knowledge of what type of ONS they should prescribe for a patient who had difficulty swallowing and repeatedly had pneumonia, and the maximum length of the overnight fast. These were important learning objectives in the revised educational intervention. When food enrichment and dietary counselling are insufficient, prescribing oral nutritional supplements correctly can be required to meet personal needs, improve nutritional status and achieve the goals of good nutritional care, caring actions that are needed and requested in clinical practice (Volkert *et al.* 2019).

The comparison between the revised and the original interventions found no differences in improvements in most areas of perceived challenges and actions related to nutritional care. However, responses to statements concerning the identification of patients at risk of undernutrition and conducting dietary assessment when suspecting undernutrition had improved less after the revised intervention. Reasons for this could be that the district nurses participating in the revised intervention were younger and had less professional experience, both in health care and as district nurses, and had more recently completed their specialist education, while the district nurses who received the original intervention had longer experience. This result may address the fact that less experienced district nurses often lack a mentor (colleague) to discuss diverse nutritional dilemmas with, together with an increased need from increasingly older adults living at home. An additional revision of the educational intervention, with a more holistic, person-centered and active approach, expanding time for interaction, reflection and discussions, may open up for further improvements.

Factual knowledge improved more after the revised intervention than after the original educational intervention in half of the areas assessed, including the type of ONS that should be prescribed and the maximum length of overnight fast. These positive results support the quality of nutritional care provided by district nurses and clarify the importance of following national guidelines. Dent *et al.* (2023) note that to gain adherence, upcoming guidelines for nutritional care need to be current, address barriers, facilitate feasibility, and be user-friendly. However, the integration of nutritional guidelines in care is inadequate, and gaps between practice and the latest evidence have been identified (Dent *et al.*, 2023).

This revised educational intervention facilitated participants by extending knowledge to become more in-depth and applicable for practice. Moreover, to increase evidence in nutritional care, intended learning outcomes, learning activities, and assessments were aligned according to the constructive alignment theory (Biggs *et al.*, 2022). In this way, the continuing education was adapted to the professionals’ work, with patient cases relevant to primary health care, initiated by the ConPrim model (Berggren *et al.*, 2016). Considering the positive improvements in perceived and factual knowledge about nutritional care, the construction behind the educational intervention should be transparent.

### Methodological considerations

The current study has several strengths. The questions and statements in the study-specific questionnaire have been pre-tested and evaluated in a previous study (Samuelsson *et al.*, 2019). Statements about perceived challenges and actions that related to nutritional care had Likert-type response options, and questions about factual knowledge had multiple choice response options, one of which was correct. This prevented participants from answering ‘do not know’ or ‘no opinion’ and maximized the gathering of responses that could ensure a more robust interpretation of the results. A total of 91% of the participants responded to the questionnaire before and after the course, which shows a low dropout rate. Participating district nurses had the opportunity to learn from and with each other in the reflective discussions during the course and to contribute with their diverse experiences and perspectives from a variety of socio-demographic and geographical areas.

Despite these strengths, the use of the study-specific self-reported questionnaire may be a limitation. At the time of the study, neither a validated questionnaire nor a supporting educational structure suitable for primary health care were available. An additional limitation of the present study was the quasi-experimental pre- and post-test design in the evaluation of the revised intervention, which only enabled us to investigate within-group changes in perceived challenges and actions related to nutritional care and factual knowledge. A further limitation is that the statistical tests for using a composite measure are lacking, preventing the assessment of progress for individual survey questions. Possible disadvantages with testing each question individually include an increased risk of type I errors and reduced statistical power. No power calculation to estimate sample size calculation was conducted since the selection was limited to a number of enlisted participants and the scope of the course. We can, therefore, not ensure that the study had sufficient statistical power to draw meaningful conclusions and avoid type II errors (false negatives). However, over a hundred participants is a common number for achieving strength (Polit & Beck, 2021).

Participants in the revised and original educational interventions were included on different occasions. Moreover, the number of participants and participant characteristics (i.e., age and experience) differed between the groups. These factors must be kept in mind when interpreting the results of the comparative analyses. However, the result showing that the district nurses in the revised intervention learned more in depth about how to meet older person’s individual needs for nutritional care strengthens the use of constructive alignment with adapted learning activities. Thus, the pedagogical support increases in-depth knowledge with benefits for older adults’ well-being and health.

## Conclusion

In this revised educational intervention, the district nurses’ assessment of perceived challenges and actions related to nutritional care and factual knowledge improved, especially concerning the type of ONS that they should prescribe to older adults and the maximum length of overnight fast. Participants also gained more in-depth knowledge about nutritional counselling, food adaptation, and how to prescribe ONS in an individually tailored way compared to the original educational intervention. Moreover, district nurses found it less difficult to know how meals should be adapted to patients’ individual needs in different palliative phases of the disease. This in-depth knowledge supports usability in clinical practice although there may be a discrepancy between what is known and what is used in daily practice. We need, therefore, more evidence for if and how this knowledge is implemented in clinical practice through the use of registry studies, RCT studies, and qualitative studies to capture benefits for older adults’ health and well-being.

### Relevance to clinical practice

After the educational intervention, district nurses have the prerequisite knowledge to safely provide oral nutritional supplements and advice about food/fluid fortification or enrichment and give dietary counselling and education to older adults. This subject-specific intervention will continue to be used and will be offered to doctors and nurses working in primary health care since they are well placed to lead the essential processes for the nutritional care of older adults.
